# Supplementary material for: A quantitative model predicts how m6A reshapes the kinetic landscape of nucleic acid hybridization and conformational transitions
Source: Nat Commun. 2021 Aug 31;12:5201. doi: 10.1038/s41467-021-25253-8 (PMC8408185; doi:10.1038/s41467-021-25253-8)
Supplement: Supplementary file 3 — Reporting Summary [file 41467_2021_25253_MOESM3_ESM.pdf]

Corresponding author(s): Hashim M. Al-Hashimi

Last updated by author(s): 7/13/2021

Reporting Summary

Nature Research wishes to improve the reproducibility of the work that we publish. This form provides structure for consistency and transparency in reporting. For further information on Nature Research policies, see our Editorial Policies and the Editorial Policy Checklist.

Statistics

For all statistical analyses, confirm that the following items are present in the figure legend, table legend, main text, or Methods section.

n/a	Confirmed	
☐	☒	The exact sample size (*n*) for each experimental group/condition, given as a discrete number and unit of measurement
☐	☒	A statement on whether measurements were taken from distinct samples or whether the same sample was measured repeatedly
☒	☐	The statistical test(s) used AND whether they are one- or two-sided<br>*Only common tests should be described solely by name; describe more complex techniques in the Methods section.*
☐	☒	A description of all covariates tested
☐	☒	A description of any assumptions or corrections, such as tests of normality and adjustment for multiple comparisons
☐	☒	A full description of the statistical parameters including central tendency (e.g. means) or other basic estimates (e.g. regression coefficient) AND variation (e.g. standard deviation) or associated estimates of uncertainty (e.g. confidence intervals)
☒	☐	For null hypothesis testing, the test statistic (e.g. F, t, r) with confidence intervals, effect sizes, degrees of freedom and P value noted<br>*Give P values as exact values whenever suitable.*
☒	☐	For Bayesian analysis, information on the choice of priors and Markov chain Monte Carlo settings
☒	☐	For hierarchical and complex designs, identification of the appropriate level for tests and full reporting of outcomes
☐	☒	Estimates of effect sizes (e.g. Cohen's *d*, Pearson's *r*), indicating how they were calculated

Our web collection on statistics for biologists contains articles on many of the points above.

Software and code

Policy information about availability of computer code

Data collection	AMBER 16.0<br>TopSpin 3.2<br>MELTING 5.2<br>deMon2k 5.0
Data analysis	NMRPipe 8.6<br>Sparky 3.1<br>AMBER 16.0<br>VMD 1.9.3<br>AFNMR 1.2<br>MEAD 2.2.10<br>PyMOL 1.5<br>DSSR 1.6<br>TopSpin 3.2<br>PyMOL 1.5<br><br>Custom in-house Python scripts for kinetic simulation/prediction and CEST simulation/fitting are available at https://github.com/alhashimilab/m6A_hybridization_kinetics (DOI: 10.5281/zenodo.5099562).<br><br>The force field parameters for m6A and m62A used in MD simulations and PDB files of these structures that were submitted to the DFT calculations are provided at https://github.com/alhashimilab/m6A_ES (DOI: 10.5281/zenodo.5099581).<br><br>The results of PDB (RCSB Protein Data Bank) survey for singly H-bond AU bps are provided at https://github.com/alhashimilab/Singly_HB_AU

(DOI: 10.5281/zenodo.5099558).

For manuscripts utilizing custom algorithms or software that are central to the research but not yet described in published literature, software must be made available to editors and reviewers. We strongly encourage code deposition in a community repository (e.g. GitHub). See the Nature Research guidelines for submitting code & software for further information.

Data

Policy information about availability of data

All manuscripts must include a data availability statement. This statement should provide the following information, where applicable:

- Accession codes, unique identifiers, or web links for publicly available datasets
- A list of figures that have associated raw data
- A description of any restrictions on data availability

The data that support this study are contained in the published article (and its Supplementary Information).

The NMR R1ρ, CEST and imino exchange data as well as kinetic simulation and prediction results generated in this study are provided at https://github.com/alhashimilab/m6A_hybridization_kinetics (DOI: 10.5281/zenodo.5106694).

The force field parameters for m6A and m62A used in MD simulations and PDB files of these structures that were submitted to the DFT calculations are provided at https://github.com/alhashimilab/m6A_ES (DOI: 10.5281/zenodo.5099581).

The results of PDB (RCSB Protein Data Bank) survey for singly H-bond AU bps are provided at https://github.com/alhashimilab/Singly_HB_AU (DOI: 10.5281/zenodo.5099558).

The DNA m6A sites used in this study were reported in a prior study. See supplementary Table 5 of the cited paper (https://www.nature.com/articles/nature17640#Sec26).

Source data are provided with this paper.

Field-specific reporting

Please select the one below that is the best fit for your research. If you are not sure, read the appropriate sections before making your selection.

☒ Life sciences ☐ Behavioural & social sciences ☐ Ecological, evolutionary & environmental sciences

For a reference copy of the document with all sections, see nature.com/documents/nr-reporting-summary-flat.pdf

Life sciences study design

All studies must disclose on these points even when the disclosure is negative.

Sample size	Determination of the sample size was not necessary for in vitro and quantitative NMR RD experiments.<br>We predicted the effect of m6A on DNA hybridization kinetics for 5,951 m6A sites in the mouse genome. These m6A sites were reported in a prior study (Wu et al, Nature 2016). See supplementary Table 5 of the cited paper (https://www.nature.com/articles/nature17640#Sec26).
Data exclusions	No data was excluded from this study.
Replication	RNA samples were prepared through solid-state synthesis >2 times and produced similar results from NMR experiments.
Randomization	Not applicable. No randomization was needed in this study because NMR experiments are in vitro and quantitative.
Blinding	Not applicable. No blinding was used in this study, because objective and quantitative NMR experiments were performed and samples were treated equally.

Reporting for specific materials, systems and methods

We require information from authors about some types of materials, experimental systems and methods used in many studies. Here, indicate whether each material, system or method listed is relevant to your study. If you are not sure if a list item applies to your research, read the appropriate section before selecting a response.

Materials & experimental systems

n/a	Involved in the study
☒	☐ Antibodies
☒	☐ Eukaryotic cell lines
☒	☐ Palaeontology and archaeology
☒	☐ Animals and other organisms
☒	☐ Human research participants
☒	☐ Clinical data
☒	☐ Dual use research of concern

Methods

n/a	Involved in the study
☒	☐ ChIP-seq
☒	☐ Flow cytometry
☒	☐ MRI-based neuroimaging
